# Nonlinear frequency analysis of COVID-19 spread in Tokyo using empirical mode decomposition

**DOI:** 10.1038/s41598-022-06095-w

**Published:** 2022-02-09

**Authors:** Ran Dong, Shaowen Ni, Soichiro Ikuno

**Affiliations:** 1 School of Computer Science, Tokyo University of Technology, Tokyo, 192-0982 Japan; 2 Graduate School of Systems and Information Engineering, University of Tsukuba, Ibaraki, 305-8577 Japan

**Keywords:** Computational science, Scientific data, Health policy, Viral infection, Epidemiology

## Abstract

Empirical mode decomposition (EMD) was adopted to decompose daily COVID-19 infections in Tokyo from February 28, 2020, to July 12, 2021. Daily COVID-19 infections were nonlinearly decomposed into several monochromatic waves, intrinsic mode functions (IMFs), corresponding to their periodic meanings from high frequency to low frequency. High-frequency IMFs represent variabilities of random factors and variations in the number of daily PCR and antigen inspections, which can be nonlinearly denoised using EMD. Compared with a moving average and Fourier transform, EMD provides better performance in denoising and analyzing COVID-19 spread. After variabilities of daily inspections were weekly denoised by EMD, one low-frequency IMF reveals that the average period of external influences (public health and social measures) to stop COVID-19 spread was 19 days, corresponding to the measures response duration based on the incubation period. By monitoring this nonlinear wave, public health and social measures for stopping COVID-19 spread can be evaluated and visualized quantitatively in the instantaneous frequency domain. Moreover, another low-frequency IMF revealed that the period of the COVID-19 outbreak and retreat was 57 days on average. This nonlinear wave can be used as a reference for setting the timeframe for state of emergency declarations. Thus, decomposing daily infections in the instantaneous frequency domain using EMD represents a useful tool to improve public health and social measures for stopping COVID-19 spread.

## Introduction

The coronavirus disease of 2019 (COVID-19) is spreading around the globe and significantly influencing the restaurant industry and travel industry. Many studies have employed various methods to analyze the spread of COVID-19. For example, Farzanegan et al.^1^ conducted an empirical analysis investigating case fatality rates between different countries. In contrast, Cinarka et al.^2^ analyzed Google searches interested in COVID-19 to track its spread using dynamic conditional correlation analysis. Most studies are only based on the time domain and linear models. Alternatively, Iftimi et al.^3^ investigated the relationship between daily COVID-19 infections in the first and second waves in Reus, Spain, and found that COVID-19 spread was periodic with the implementation of public health measures. Therefore, an analysis method in the frequency domain is desirable when dealing with complex nonlinear data such as COVID-19 daily infections.

Huang et al.^4^ proposed empirical mode decomposition (EMD) to nonlinearly decompose composite signals collected in the real world. Using EMD, these waves can be decomposed into several pseudo monochromatic waves called intrinsic mode functions (IMFs) and a residual called a trend. Since the IMF is a pseudo monochromatic wave, the Hilbert transform (HT) is employed to obtain the instantaneous frequency and amplitude^5^; applying the HT to decomposed IMFs enables the analysis of the composited signal in the instantaneous frequency domain^6^. As a result, EMD is widely applied in nonlinear analyses in the instantaneous frequency domain. Thus, for analyzing the nonlinear spread of COVID-19, EMD is more suitable than using a moving average and a Fourier transform (FT).

Various research fields related to COVID-19 have also adopted EMD. Mahata et al.^7^ showed that EMD and its Hilbert spectrum could be adopted to analyze the stock market crash caused by COVID-19. In this case, EMD showed its highest performance when dealing with nonlinear data, revealing characteristics of the stock market crash during the COVID-19 pandemic as events unfolded. Hasan et al.^8^ proposed a method employing IMFs decomposed by EMD to train a neural network, predicting the spread of COVID-19. Qiang et al.^9^ examined the spread of COVID-19 in Pakistan using EMD, and demonstrated that decomposed IMFs could be used to analyze COVID-19 spread based on an autoregressive integrated moving average (ARIMA) model. Liu et al.^10^ also conducted research analyzing COVID-19 spread using EMD based on the ARIMA model. However, all of these studies only used EMD based on neural networks or statistical models. There has been no explanation or discussion about the meaning of each decomposed IMF regarding regional circumstances.

Since public health measures and social restrictions aimed at stopping the spread of COVID-19 vary between countries and regions, some studies have compared regional differences. Mishra et al.^11^ compared public health measures implemented by the United Kingdom, Sweden, and Denmark and demonstrated that COVID-19 spread differed due to unique measures that were applied in each region. Fraser et al.^12^ studied COVID-19 spread in Japan based on social ties and revealed that COVID-19 cases differed among 47 prefectures. Thus, the spread of COVID-19 varies between different areas that are under distinct circumstances. Meanwhile, Watanabe et al.^13^ suggested that during the COVID-19 outbreak in Japan, there were intervention effects due to the public health measures taken by the government and information effects from the social measures that changed people’s behaviors. However, there has been no research of daily infections in Tokyo using EMD regarding regional circumstances based on intervention and information effects. Therefore, in this paper, EMD was adopted to analyze daily COVID-19 infections in Tokyo (the capital city of Japan that has experienced four waves since the original severe acute respiratory syndrome coronavirus 2 (SARS-CoV-2) was detected in Japan) with respect to intervention effects and information effects. We demonstrated meanings of social activities in the instantaneous frequency domain to reveal new knowledge regarding the COVID-19 spread. Additionally, we provide quantitative indicators to guide policy developments for future public health and social measures.

This study analyzed daily COVID-19 infections in Tokyo from February 28, 2020, to July 12, 2021. This period was chosen because the delta variant of SARS-CoV-2 has a different spread pattern in Tokyo due to its strong infectivity^14^, compared to the alpha variant, which was the primary variant in Tokyo until June 2021^15^ and which has almost the same infectivity as the original variant. Thus, for simplicity, our research only focuses on COVID-19 daily infections in Tokyo before the delta variant became dominant. Additionally, the frequency used in this paper is defined as cycles per day as EMD was adopted to analyze daily infections.

## Methods

In this section, we briefly introduce empirical mode decomposition (EMD). EMD decomposes a real-world signal into several pseudo monochromatic waves, called intrinsic mode functions (IMFs). After decomposing the signal into each IMF, their instantaneous frequencies and amplitudes were obtained by Hilbert transform (HT) based on the analytic signal. Therefore, we briefly review analytic signal, HT, and EMD.

### Analytic signals

Analytic signals are widely employed in signal processing research fields. An analytic signal has two parts that form a complex plane: One part is a real part, while the other is an imaginary part orthogonal to the real part. An analytic signal is defined by the following (1):

1 $$\begin{aligned} z(t)=z_r (t)+iz_i (t) = a(t)e^{i\theta {(t)}} \end{aligned}$$

where $z_r(t)$ denotes the real part and $z_i(t)$ denotes the imaginary part. As shown in (1), amplitude *a*(*t*) and phase $\theta (t)$ change as time passes. Therefore, the instantaneous amplitude *a*(*t*), the instantaneous phase $\theta (t)$, and the instantaneous frequency $\omega (t)$ can be calculated based on an analytic signal by (2):

2 $$\begin{aligned} a(t)=\sqrt{z_r^2(t) + z_i^2 (t)},\quad \theta {(t)}=\arctan {\left( \frac{z_i (t)}{z_r (t)}\right) },\quad \omega (t)=\frac{d}{dt}\arctan {\left( \frac{z_i (t)}{z_r (t)}\right) } \end{aligned}$$

### Hilbert transform

As only the real part of an analytic signal can be collected in the real world, the imaginary part must be calculated based on the real part (the observed signal). HT can convert the real part $z_r(t)$ into its imaginary part $z_i(t)$ by assuming $z_r(t)$ is a monochromatic wave $a(t)\cos (\omega (t)t)$. Hence, HT, as defined by (3), calculates the imaginary part $z_i(t)$ from the real part $z_r(t)$^5^.

3 $$\begin{aligned} z_i(t)=\frac{1}{\pi } {\mathrm{PV}} \int ^{\infty }_{-\infty }\frac{z_r(\tau )}{(t-\tau )} d\tau =\frac{1}{\pi {t}}*z_r(t) \end{aligned}$$

Here, PV represents the Cauchy principal value. After calculating $z_i(t)$ by HT, the analytic signal shown in (1) is obtained. Then, the instantaneous frequency and amplitude of the observed signal can be determined by (2).

### Empirical mode decomposition

To calculate an imaginary part, HT requires that the signal be a monochromatic wave $a(t)\cos (\omega (t)t)$. However, signals observed in the real world are usually composite waves. To apply HT to composite waves, EMD decomposes these waves into several pseudo monochromatic waves, called intrinsic mode functions (IMFs), and a residual called a trend^6^. Hence, a composite wave observed in the real world *x*(*t*) can be defined by (4):

4 $$\begin{aligned} x(t)=\sum ^n_{i=1}c_i(t) + r(t) \end{aligned}$$

where ${\sum \nolimits ^n_{i=1}c_i(t)}$ is the set of IMFs, and *r*(*t*) is the residual. The definition of IMF is as follows:

- The number of signal extrema equals zero crossings, or the difference is 1.
- The average value of the two extreme envelopes made by the maximum and minimum is 0 for any *t*.

The trend is empirically defined by the following^16^:

- A trend is a monotonic function or a function with at most one extremum, intrinsically fitting the data with a given span.
- Detrending is an operation that removes the trend, and variability is the residue removed trend within a given span.

Based on these definitions of IMFs and trends, Huang et al.^4,6^ proposed a one-variable EMD, as shown in Algorithm (1). 
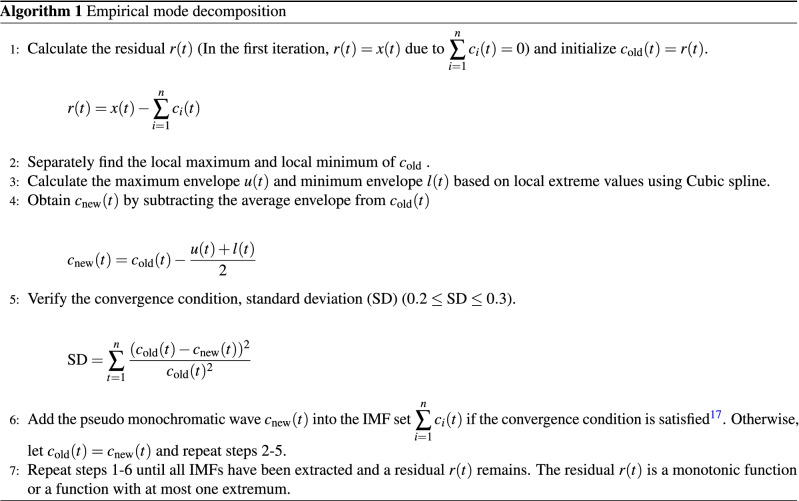


After decomposing the observed signal into several IMFs using EMD, their instantaneous frequencies and instantaneous amplitudes can be obtained by applying HT to each IMF. This entire process is also called the Hilbert-Huang transform (HHT). The frequency components of the original signal *x*(*t*) can be expressed by HHT, as shown in (5):

5 $$\begin{aligned} x(t)={\mathrm{Re}}\left\{ \sum ^n _{j=1}a_j(t)\exp \left[ i\int {\omega _j{(t)}dt}\right] \right\} \end{aligned}$$

while the frequency components of the original signal *x*(*t*), obtained by Fourier transform (FT), are shown in (6):

6 $$\begin{aligned} x(t)={\mathrm{Re}}\left[ \sum ^n _{j=1}a_j{\exp (i\omega _j{t})}\right] \end{aligned}$$

By comparing (5) and (6), FT linearly decomposes a signal with constants $a_j$ and $\omega _{j}$ while EMD nonlinearly decomposes data into finite IMFs with variables $a_j(t)$ and $\omega _{j}(t)$. Thus, for nonlinear data such as the spread of COVID-19, which is influenced by various nonlinear factors, frequency analysis using EMD is more suitable than FT.

In addition, Niu et al.^18^ proposed a weighted average frequency algorithm (WAFA) that can smooth the instantaneous frequencies of each IMF by treating instantaneous amplitudes as weights to reduce decomposition errors. Thus, WAFA was employed in this study to smooth IMFs and obtain averaged frequencies of each IMF.

## Results

We adopted EMD to analyze daily COVID-19 infections in Tokyo, Japan. First, we demonstrate the decomposition results of each IMF and the trend. The periodic meanings of each IMF were identified by analyzing the decomposed IMFs in the instantaneous frequency domain using HT. Next, to show that EMD can decompose COVID-19 data nonlinearly, we compared EMD outcomes with the moving average and Fourier transform approach to denoise variabilities of (1) random factors; and (2) variations in the number of daily polymerase chain reaction (PCR) and antigen inspections. Finally, after daily COVID-19 infections were denoised, the periodic meaning of other decomposed IMFs are discussed, and their applications are proposed.

### Decomposition of daily COVID-19 infections using EMD

**Figure 1:**
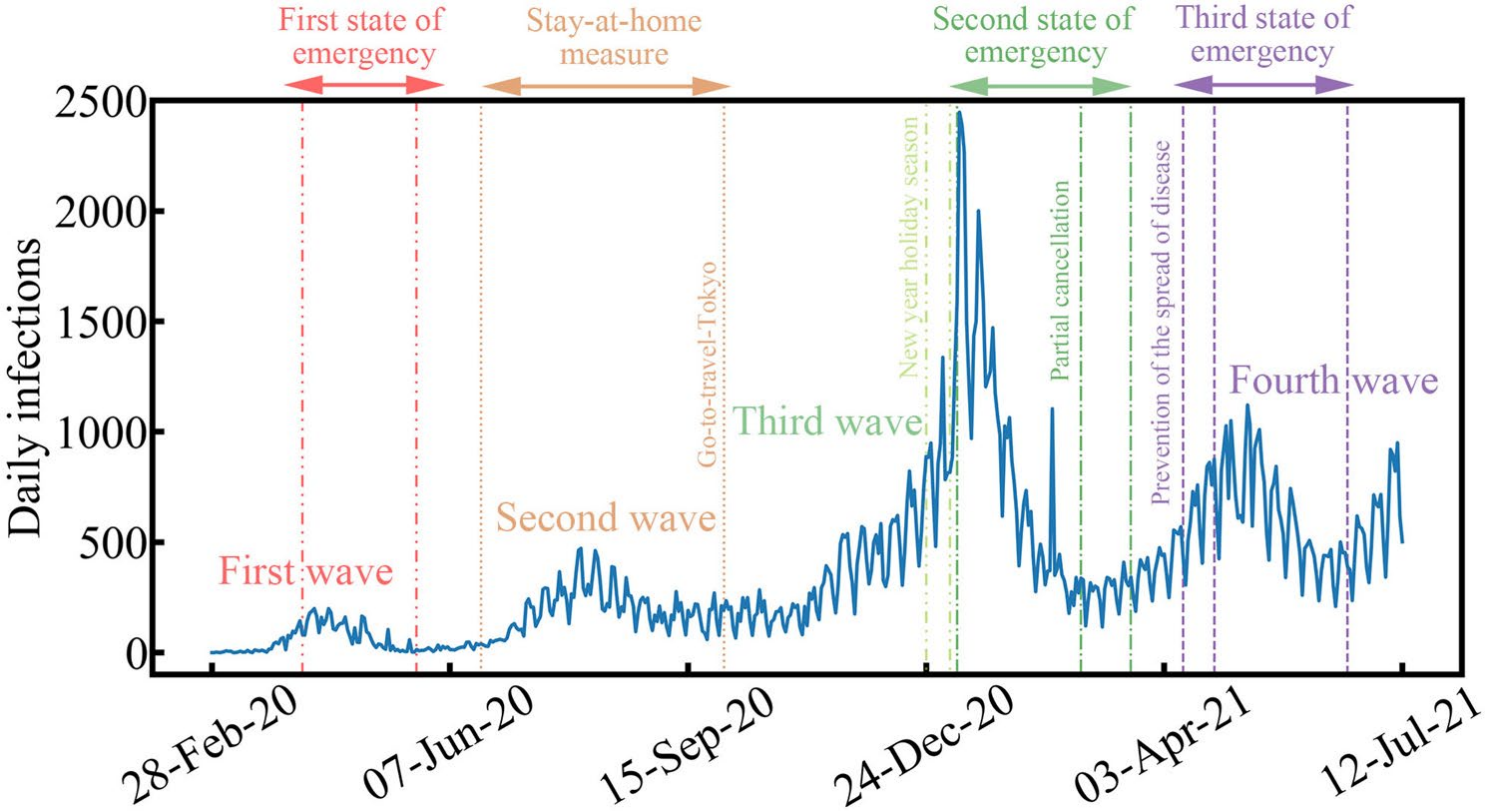
Daily COVID-19 infections in Tokyo, Japan, from February 28, 2020, to July 12, 2021.

Nonlinear frequency analysis using EMD was performed on daily COVID-19 infections in Tokyo, Japan. The COVID-19 infection data were published by the Tokyo metropolitan government^19^. The analysis included data from February 28, 2020, to July 12, 2021. It is thought that most infections during this period were the alpha variant before the delta variant became rampant. Figure 1 shows the original daily COVID-19 infections data in Tokyo. Four waves of COVID-19 spread occurred from February 28, 2020, to July 12, 2021. The data is noisy as the daily infections represent weekly changes. In addition, it can also be seen that the spread of COVID-19 was nonlinear due to external influences such as the implementation of public health and social measures to restrict the spread of COVID-19, as suggested by the World Health Organization^20^. For instance, the government of Japan issued three state of emergency periods during the timeframe of the analysis. As shown in Fig. 1, the state of emergency measures suppressed COVID-19 spread, as all waves decreased during that period. Other measures, including partial cancellation and the prevention of the spread of disease initiatives, were also used to control COVID-19 spread gradually. Alcohol could not be served in restaurants during these periods, and admission to department stores was restricted. These events can be thought of as public health measures taken by the government. On the other hand, social measures are related to people’s choice to voluntarily stay at home in response to media reports and a stay-at-home measure announced by the Tokyo metropolitan government. As shown in Fig. 1, the spread of COVID-19 was controlled under the stay-at-home measure without the need for a state emergency in the second wave.

**Figure 2:**
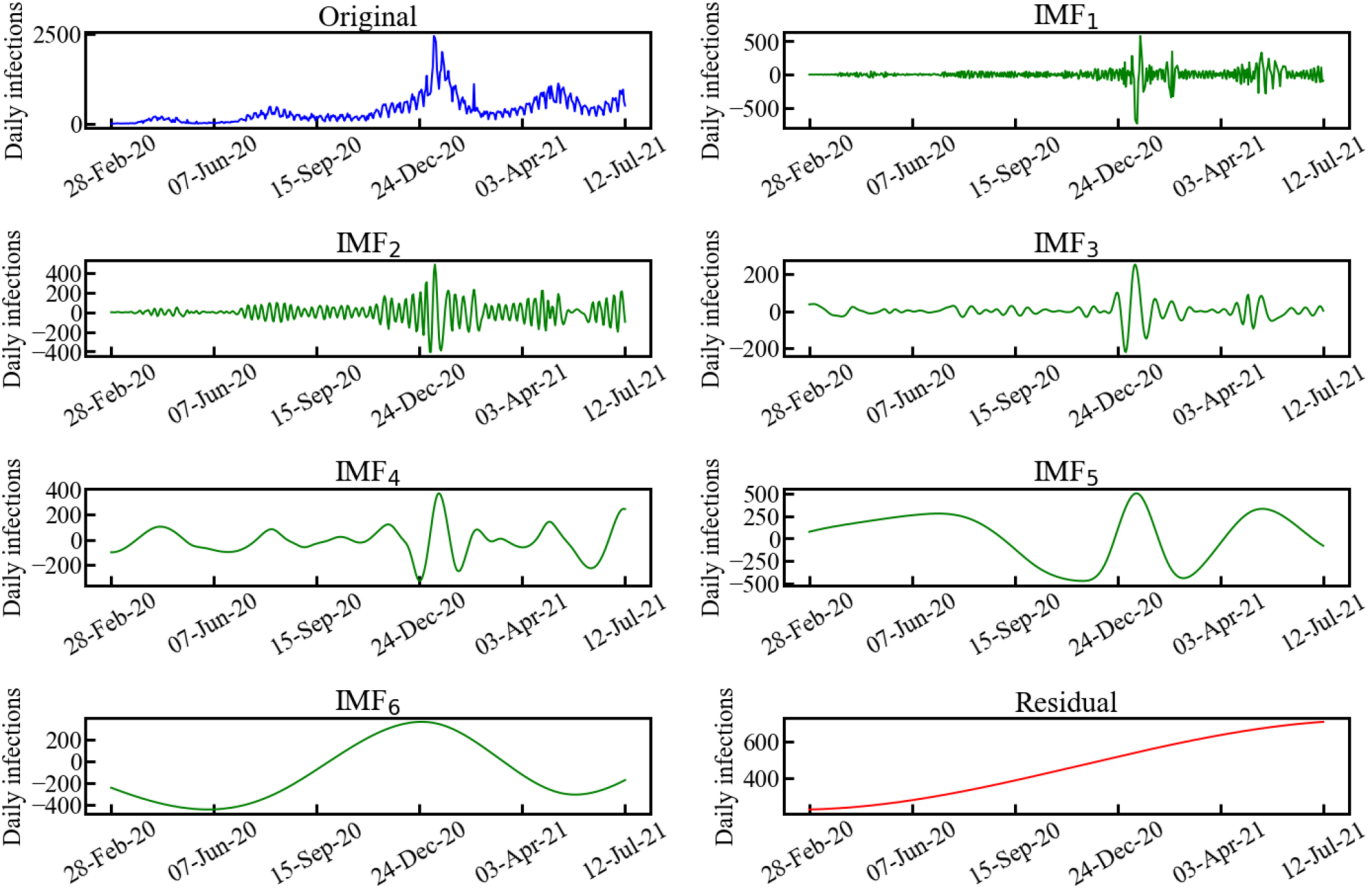
Daily COVID-19 infections in Tokyo, Japan, nonlinearly decomposed by EMD.

To analyze the nonlinear spread of COVID-19, EMD was adopted to decompose the data into several IMFs, corresponding to distinct periods that could be attributed to the public health and social measures implemented by the government and media. Figure 2 shows the decomposition results of daily COVID-19 infections by EMD. The original data were decomposed into six IMFs from high frequency (IMF$_1$) to low frequency (IMF$_6$) and a trend. The trend suggests that the spread of COVID-19 is still progressing. To reveal the meaning of the decomposed IMFs (pseudo monochromatic waves), we applied HT to each IMF to obtain their instantaneous frequencies and amplitudes.

**Figure 3:**
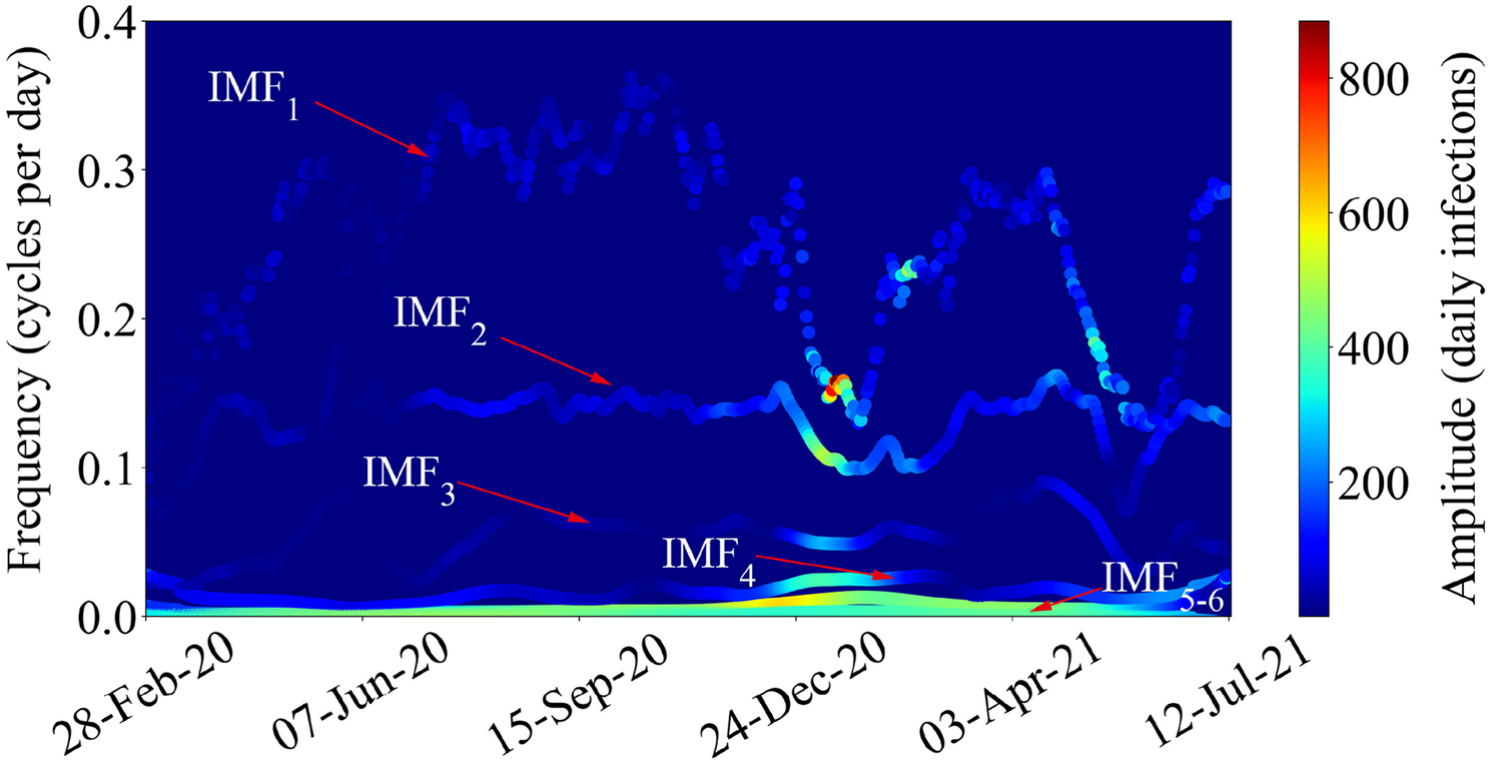
The Hilbert spectrum of each decomposed IMF in the instantaneous frequency domain.

Figure 3 shows the Hilbert spectrum of daily COVID-19 infections in Tokyo, Japan, obtained by applying HT to each IMF shown in Fig. 2. As shown in the spectrum, IMF$_1$ changes rapidly from 0.35 to 0.14 cycles per day while IMF$_2$ is around 0.128 cycles per day in the high instantaneous frequency domain. IMF$_3$ changes from 0.8 to 0.2 cycles per day, while IMF$_4$ is around 0.017 cycles per day. Low-frequency variabilities are represented by IMF$_{5}$ and IMF$_{6}$ (which have larger amplitudes than high-frequency variabilities); they can be treated as a part of the residual to reform the original data. All IMFs show high amplitude around the new year, evidencing the impact of the third wave.

To provide intuitive meanings for each decomposed IMF in the spread of COVID-19, WAFA was applied to take an average frequency of the series of events. Table 1 shows the average frequencies and average periods ± standard deviation (SD) with corresponding periodic meanings. SD was adopted to calculate the distribution of instantaneous frequencies decomposed by EMD. The lower the SD compared to the average period, the more the pattern would appear at a constant frequency. For instance, the pattern of IMF$_2$ was most stable among all IMFs. The average period of IMF$_1$ was 4.5 days (SD $\pm \; {1.312}$ days), indicating that IMF$_1$ was noisy and unstable compared to other IMFs. As demonstrated by the Tokyo metropolitan government, the number of PCR and antigen inspections was different for each day on a weekly cycle^19^. Meanwhile, the average period of IMF$_2$ was 7.8 days, indicating that IMF$_2$ corresponds to variability due to the variations in the number of PCR and antigen inspections. Thus, these two IMFs can be considered as noise to be removed in trend analysis of the COVID-19 outbreak. The average period of IMF$_3$ was 19.0 days. It was related to the external measures implemented to stop the spread of COVID-19, caused by the intervention effects and information effects of the public health measures and social measures, receptively. The average period of IMF$_4$ was 57 days. It represents the period of the COVID-19 outbreak and retreat.

Since detectable lowest frequency depends on the time duration of analysis, the lower frequency IMFs than the COVID-19 outbreak and retreat will also be decomposed when analyzing more extended data. In this research, we analyzed COVID-19 spread from February 28, 2020, to July 12, 2021 (501 days). Thus, the detectable lowest frequency by EMD is 0.002 (1/501) cycles per day, which was the average frequency of IMF$_6$, and the average frequency of IMF$_5$ was 0.006 (1/166) cycles per day. Consequently, IMF$_5$ and IMF$_6$ were less important when analyzing intervention and information effects during the COVID-19 outbreak and retreat in Tokyo. Thus, in this research, we only focused on discussing the periodic activity meanings of IMF$_1$ to IMF$_4$.

To demonstrate the meaning of each IMF’s periodic activity shown in Table 1, we discuss the IMFs based on theories and experiments. As shown in Fig. 3, although the average period of IMF$_1$ was 4.5 days, its frequency changes dramatically compared to other IMFs. Additionally, according to previous research^6^, IMF$_1$ had higher white noise than other low-frequency modes because EMD decomposes nonlinear modes from high frequency to low frequency. Thus, IMF$_1$ can be thought of as variability due to various random factors.

The daily PCR and antigen inspections are also published by Tokyo metropolitan government^19^. To verify that IMF$_2$ was generated by variations in PCR and antigen inspections, we decomposed the number of daily PCR and antigen inspections in Tokyo using EMD. The decomposition result indicated that IMF$_2$ of daily PCR and antigen inspections had an averaged period of 7.177 days and the largest amplitude among the other decomposed IMFs. This indicates that the number of daily PCR and antigen inspections in Tokyo had a periodical pattern every week. In contrast, as shown in Table 1, IMF$_2$ that decomposed from the daily infections had an averaged period of 7.797 days with SD = 1.267. Then, the averaged period (7.177 days) of IMF$_2$ that decomposed from the daily PCR and antigen inspections was in the range of 6.530 to 9.064 days. In addition, this IMF$_2$ also had the largest amplitude among other IMFs. Thus, IMF$_2$ that decomposed from the daily infections corresponds to the variations in the number of PCR and antigen inspections. IMF$_3$ and IMF$_4$ will be discussed in detail in the following section.

**Table 1:** The average frequencies and periods of each decomposed IMF by WAFA and their periodic meanings.

IMF	Averaged frequency $\pm \; \mathrm{SD}$ (cycles per day)	Averaged period $\pm \; \mathrm{SD}$ (days)	The meaning of periodic activities
1	0.221 $\pm \;{0.064}$	4.519 $\pm \;{1.312}$	Various random factors
2	0.128 $\pm \;{0.021}$	7.797 $\pm \;{1.267}$	Variations in daily PCR and antigen inspections on a weekly cycle
3	0.053 $\pm \;{0.017}$	18.988 $\pm \;{6.112}$	External influences restricting COVID-19 spread
4	0.017 $\pm \;{0.006}$	57.171 $\pm \;{19.261}$	COVID-19 outbreak and retreat in Tokyo

### Weekly denoising of daily infections using EMD

In this study, IMF$_{1}$ and IMF$_{2}$ were deleted from the original data when analyzing the outbreak of COVID-19 as IMF$_{1}$ was random factors, and IMF$_{2}$ was caused by variations in the number of daily PCR and antigen inspections. Since the period of IMF$_{2}$ was close to one week, IMFs with longer periods were considered as weekly denoised trends.

**Figure 4:**
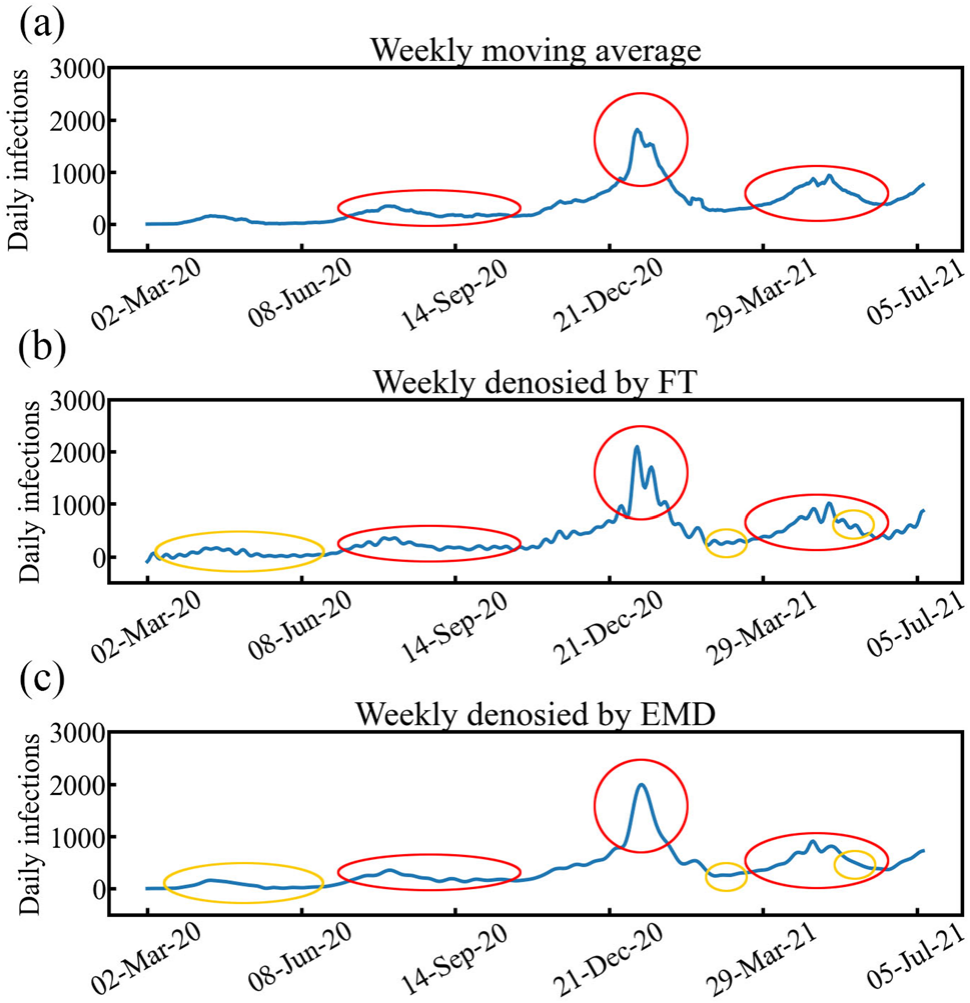
Comparison among (**a**) moving average, (**b**) FT, and (**c**) EMD in weekly denoised trends.

To demonstrate the nonlinear denoising method using EMD, we compared our results with moving average and FT, as shown in Fig. 4. In contrast to the moving average and FT methods, EMD can decompose daily infections data nonlinearly with their period properties. Figure 4a shows daily infections denoised by taking the average over a 7-day window. The moving average over a 7-day window is widely used when analyzing COVID-19 spread to remove weekly variability. As shown in Fig. 4a, weekly variability was denoised without considering any period properties. Figure 4b shows daily infections denoised weekly by FT. All of the periods that were less than seven days were removed. As shown in Fig. 4b, period properties were considered to present the periodic changes. However, as we mentioned above, FT linearly decomposes data such that the FT cannot provide periodic meanings regarding social activities when dealing with nonlinear data like the spread of COVID-19. Figure 4c shows daily infections denoised weekly by EMD, IMF$_{1}$ and IMF$_{2}$ were removed.

Although both Fig. 4b,c have periodicity, FT shows vibrations all the time as it decomposes data with constant frequency. As the yellow circles demonstrate, vibrations in daily infections denoised weekly by FT can be clearly observed, while daily infections denoised weekly by EMD has almost no vibration. It can be suspected that these frequency components obtained by FT only capture local frequency properties, while EMD can handle a global range with all COVID-19 daily infections in the frequency domain. Since social activities occur nonlinearly in the time series, EMD is more likely to decompose meaningful social activities than FT. Thus, EMD provides better explanations than other methods when analyzing the spread of COVID-19.

### Nonlinear frequency analysis using EMD

**Figure 5:**
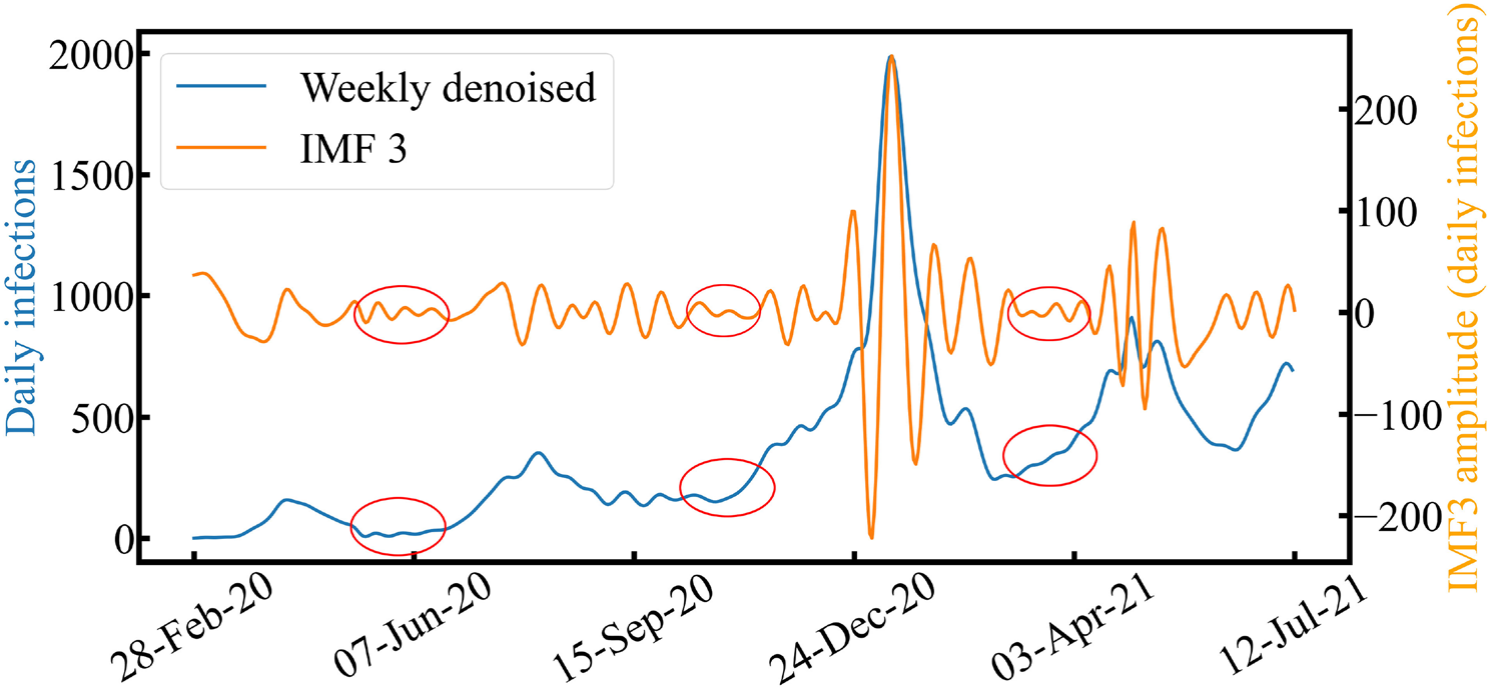
Comparison between weekly denoised trend and decomposed IMF$_3$.

Next, we employed EMD to analyze the effects of the interventions and information provided on the spread of COVID-19, which corresponds to IMF$_3$, and the COVID-19 outbreak and retreat in Tokyo, which corresponds to IMF$_4$. Figure 5 shows the results of IMF$_3$ with a weekly denoised trend. Nonlinear components of IMF$_3$ existed around the four waves, becoming smaller after each wave (red circles). This finding suggests that IMF$_3$ reflects the intervention effects and information effects described in the introduction. Nonlinear components become smaller as these effects diminish due to the cancellation of the state of emergency. The average period was 19 days, so it can be considered that information effects and intervention effects (social measures and public health measures) are implemented based on the incubation period of the SARS-CoV-2 virus. Then, the Hilbert spectrum of IMF$_3$ was employed to evaluate and visualize these effects more clearly.

To verify that IMF$_3$ was related to the effects of the interventions and information, we examined the correlations between IMF$_3$ and the public health measures and IMF$_3$ and the social measures. During the state of emergency and the prevention of disease initiatives, serving alcohol was prohibited by the Japanese government and the Tokyo metropolitan government based on their respective policies to prevent droplet infections. Since the number of people going to restaurants declined during that time, the year-on-year (2019) change in the number of views of the restaurant information website in Tokyo^21^ was taken to investigate the correlation between IMF$_3$ and the public health measures. In contrast, the COVID-19 information provided by social media increased following the outbreak of COVID-19. Consequently, people paid more attention to preventing the spread of COVID-19. In addition, according to previous research in Japan^22^, people who use social networking services maintain social distancing more than those who do not. Google provides a service called “Google Trends” that can analyze the popularity of Google searches in different regions and languages^23^. Thus, this study adopted the popularity of Google web searches for two words, “Tokyo” and “Corona” in Japanese (the most used words for obtaining information of COVID-19 in Tokyo), to investigate the correlation between IMF$_3$ and social measures.

**Figure 6:**
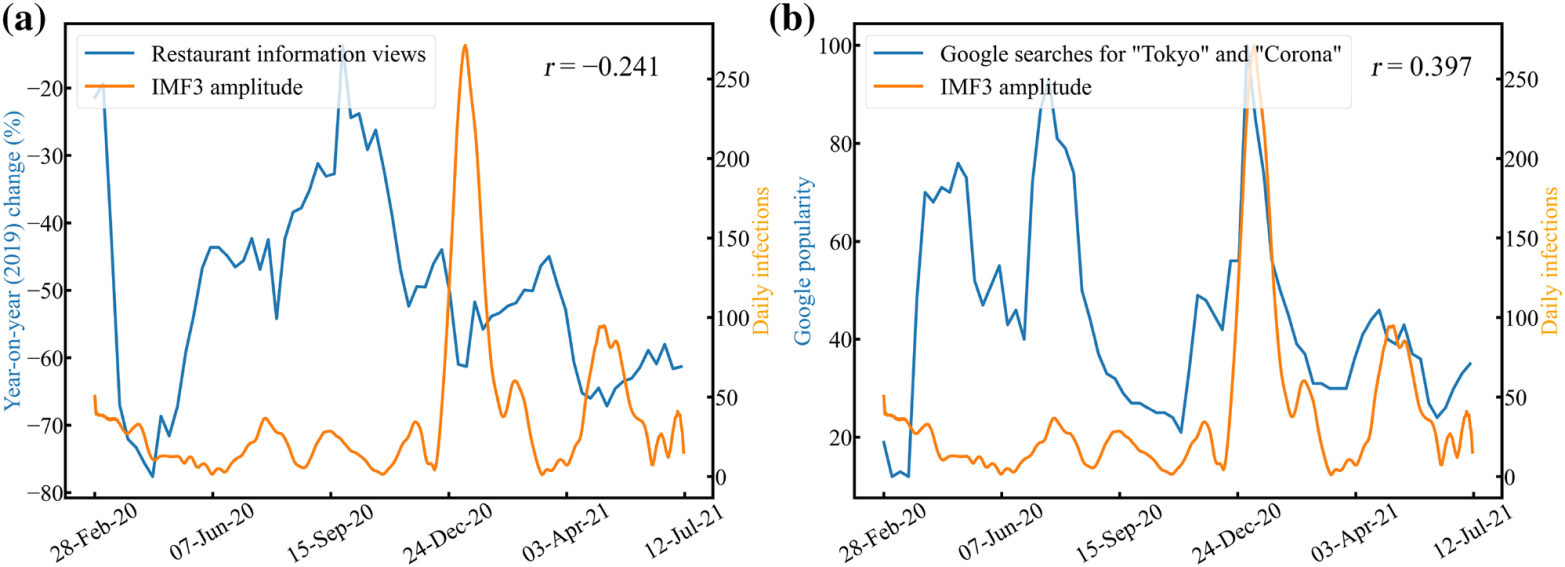
Correlations between decomposed IMF$_3$ and intervention effects (public health measures) and IMF$_3$ and information effects (social measures). (**a**) Year-on-year (2019) change in the number of restaurant information site views in Tokyo. (**b**) The popularity of Google searches for “Tokyo” and “Corona” in Japanese, obtained by Google Trends.

In the present research, we adopted the Pearson correlation coefficient to evaluate relationships between IMF$_3$ amplitude and restaurant information site views, IMF$_3$ amplitude and the popularity of Google searches. We also applied *p*-value to ensure the results were statistically significant. Since restaurant information and Google Trends only provide data after averaging in one week, we also weekly averaged IMF$_3$ amplitude to fit both of them (71 weeks). Figure 6 indicates the correlation between (a) decomposed IMF$_3$ amplitude and views of restaurant information in Tokyo, and (b) IMF$_3$ and the popularity of Google searches for “Tokyo” and “Corona.” In Fig. 6a, the blue line shows the weekly average of the year-on-year (2019) change in the number of website views. It changes rapidly during the four waves. The correlation coefficient between IMF$_3$ amplitude and restaurant information site views was $-0.241$ ($p<0.05$), indicating a weak negative correlation. This result indicates that the IMF3 amplitude increased when restaurant information views decreased and vice versa, as shown in Fig. 6a. Since people panicked during the first wave, restaurant information views declined dramatically at the beginning of the pandemic. Additionally, the PCR testing system was not ready for the COVID-19 outbreak. For these reasons, when calculating the correlation between IMF$_3$ amplitude and restaurant information site views from only the second wave to the fourth wave (61 weeks), a higher negative correlation was obtained ($r = -0.381$, $p<0.01$). Thus, when the IMF$_3$ amplitude becomes more significant, it reflects that people are keeping away from drinking and partying in restaurants, indicating that IMF$_3$ was related to the public health measures.

In Fig. 6b, the blue line shows the weekly averaged popularity of Google searches for “Tokyo” and “Corona.” A value of 100 indicates that the keyword was the most popular, while 0 indicates insufficient data for that keyword. The correlation coefficient between IMF$_3$ amplitude and Google popularity was 0.397 ($p<0.001$), indicating a positive correlation. This result indicates that the IMF3 amplitude increased when Google searches for “Tokyo” and “Corona” increased and vice versa, as shown in Fig. 6b. Since people panicked during the first wave, the web searches for “Tokyo” and “Corona” changed dramatically at the beginning of the pandemic. Therefore, when calculating the correlation coefficient between IMF$_3$ amplitude and Google popularity from only the second wave to the fourth wave (61 weeks), a higher positive correlation was obtained ($r = 0.503$, $p<0.001$). Hence, when the IMF$_3$ amplitude became more significant, people were paying more attention to the spread of COVID-19, indicating that IMF$_3$ was related to the social measures. Therefore, the correlation between the number of restaurant views and IMF$_3$ was significantly negative, especially after the second wave, whereas its correlation with the Google Trends in “Tokyo” and “Corona” searches was significantly positive. Thus, people’s behaviors were restrained by the intervention and information effects that are significantly related to IMF$_3$, indicating that IMF$_3$ can be considered as a nonlinear mode corresponding to external influence stopping the spread of COVID-19.

**Figure 7:**
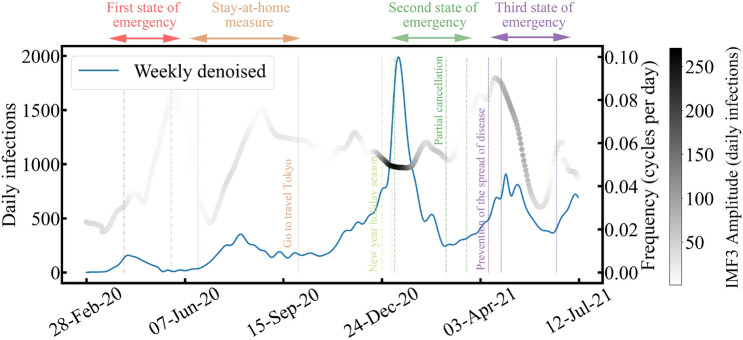
Comparison between the weekly denoised trend and the decomposed IMF$_3$ Hilbert spectrum.

Figure 7 shows the Hilbert spectrum of IMF$_3$ with daily infections of COVID-19 weekly decomposed by EMD. Social measures and public health measures are also presented in the figure. If the effects occur, the amplitude of IMF$_3$ becomes more significant. This can be clearly observed over the duration of the first state of emergency, the stay-at-home measure, the second state of emergency, and the third state of emergency. On the contrary, the amplitude disappears when these effects cease, enabling the spread of COVID-19.

Additionally, when social and public health measures were performed more frequently, the frequency of IMF$_3$ may become higher since it corresponds to the external influences aiming to stop the spread of COVID-19. The frequency of IMF$_3$ may represent the frequencies of policy implementations and media coverage on COVID-19. For example, policy implementations and media coverage were frequent at the beginning of the COVID-19 outbreak, the first and second waves. As such, this can be considered one of the reasons why the second wave could be controlled without a state of emergency. In contrast, policy implementations and media coverage were less frequent in the fourth wave as the frequency of IMF$_3$ became lower; consequently, people lost interest in COVID-19 infections. Thus, there is a possibility that information and intervention effects could be quantitatively evaluated and visualized by monitoring and analyzing IMF$_3$ in the instantaneous frequency domain.

**Figure 8:**
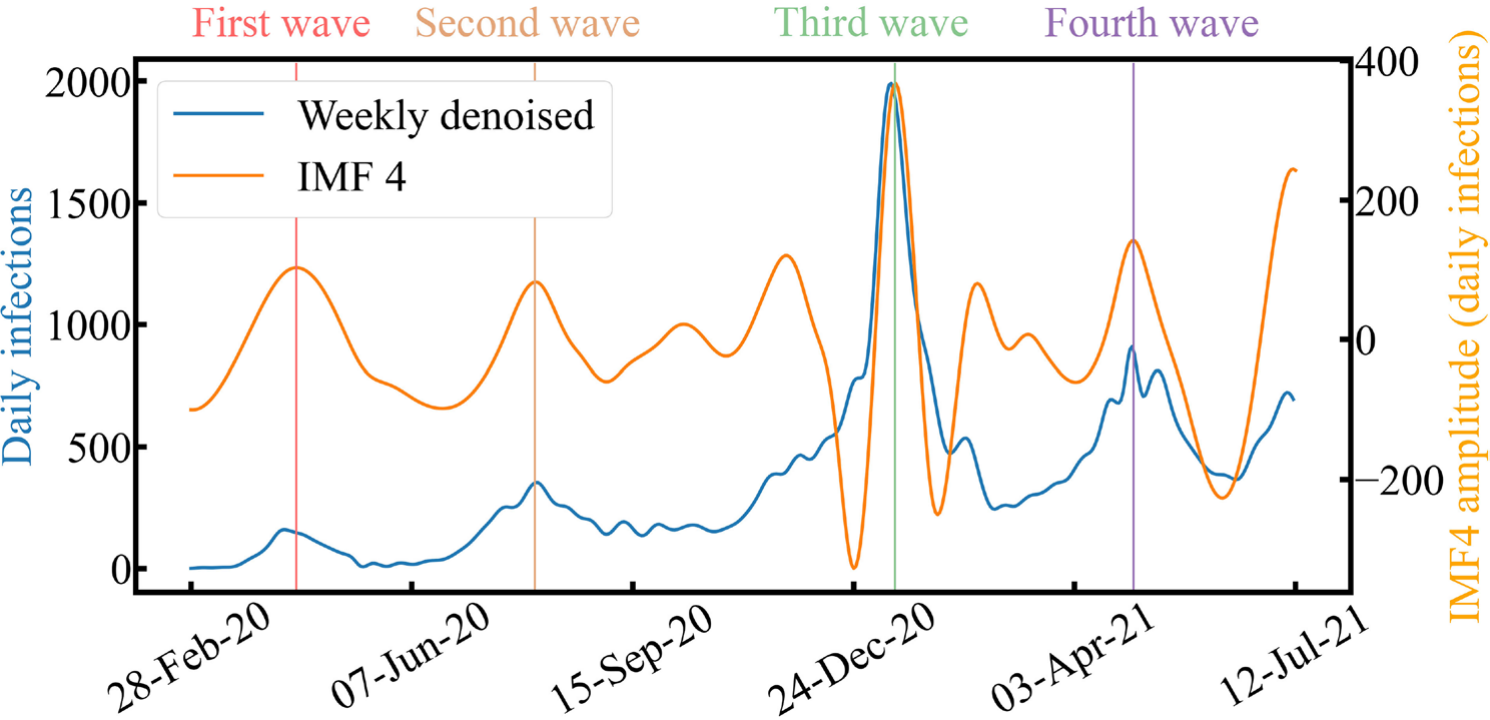
Comparison between the weekly denoised trend and decomposed IMF$_4$. Vertical lines indicate the four waves.

Figure 8 shows IMF$_4$ with daily infections decomposed weekly. The four waves were synchronized with weekly decomposed daily infections. This observation indicates the IMF$_4$ represents the period of the COVID-19 outbreak and retreat in Tokyo, with a duration of 57 days. It should be noted that there were two waves near the third wave. The third wave caused this due to its large amplitude, which other IMFs could cancel when rebuilding the original data, as shown in Fig. 4a. Since the cancellation of the state of emergency can be thought of as the end of the outbreak, the average duration of the state of emergency can be considered as the period of the COVID-19 outbreak and retreat in Tokyo. To prove that IMF$_4$ was related to the COVID-19 outbreak in Tokyo, we calculated the average duration (53 days) from the first (49 days), second (53 days), and third (56 days) state of emergency periods. On the contrary, the average period duration of IMF$_4$ was $57 \pm {19}$ days, close to the average duration of the states of emergency. Thus, IMF$_4$ represents the period of the COVID-19 outbreaks and retreats in Tokyo. Monitoring and analyzing IMF$_4$ could provide a quantitative indicator when planning the duration of the state of emergency.

## Discussion

In this paper, we adopted EMD to analyze daily COVID-19 infections in Tokyo, Japan, from February 28, 2020, to July 12, 2021. Our results showed that EMD decomposes daily infections into several IMFs, corresponding to nonlinear waves with periodic meanings of social activities.

First, our results revealed that IMF$_1$ corresponded to various random factors, while IMF$_2$ corresponded to the variations in daily PCR and antigen inspections in Tokyo since the number of inspections on weekends are low while the number of inspections on weekdays are high^19^. Our results showed that the average period of IMF$_1$ was 4.5 days, and IMF$_2$ was 7.8 days. As shown in Fig. 3, the instantaneous frequency of IMF$_2$ was nearly constant, around 0.128 cycles per day, demonstrating that the number of inspection changes on a weekly cycle. By deleting IMF$_1$ and IMF$_2$ from the original data, the trend of the COVID-19 spread in Tokyo could be visualized and analyzed more clearly. Compared with the moving average and FT methods, EMD showed better performance in dealing with nonlinear data regarding social activities during the COVID-19 outbreak.

Second, our study also demonstrated that IMF$_3$ corresponded to external influences aiming to stop the spread of COVID-19. In Japan, intervention effects were related to public health measures implemented by the government, and information effects related to people’s behaviors responding to information about the pandemic^13^. The key to stopping the spread of COVID-19 was not strong measures but rather providing appropriate information to encourage people to change their behaviors^24^. Thus, there are external influences (intervention effects and information effects) acting to gradually and continually stop the spread of COVID-19. As shown in Fig. 7, the amplitude increases when the intervention effects and information effects are ongoing. The first wave was controlled by the intervention effects of the public health measures, such as the state of emergency. After that, the amplitude of IMF$_3$ diminishes, indicating that people’s behaviors returned to normal. The second wave was controlled by information effects when daily infections were increasing. According to previous research^25^, COVID-19 infections are mainly caused by human mobility. Human mobility behaviors decreased by about 30% in Tokyo during the second wave due to the stay-at-home measure^26^. Thus, the stay-at-home measure was effective because people were still paying attention to news about the virus at that time, demonstrated by the amplitude of IMF$_3$. However, the government instigated the “Go-to-travel-Tokyo” initiative, a campaign to encourage people to travel to Tokyo, which started on October 1, 2021. Although go-to-travel-Tokyo was discontinued later, people might no longer pay attention to guidance about stopping COVID-19 spread when the third and fourth waves occurred. Consequently, the second and third states of emergency were announced, and IMF$_3$ shows that more powerful external influences (larger amplitude) were implemented to stop the spread of COVID-19, compared with the first and second waves. In addition, to verify that IMF$_3$ corresponded to external influences stopping the spread of COVID-19, we examined the correlations between IMF$_3$ and restaurant information views (intervention effects) and the popularity of Google searches for “Tokyo” and “Corona” (information effects). Although the correlation coefficients were not strong (as there were too many factors influencing the spread of COVID-19, such as higher temperatures and more intense UV radiation in summer which are likely to support public health measures^27^), our results revealed that IMF$_3$ had relevance with both intervention effects and information effects. Additionally, the frequency of IMF$_3$ became smaller during the fourth wave compared to the third wave. This may suggest that people and governments were paying less attention to stopping the spread of COVID-19 during the fourth wave compared to the third wave (but they still paid some attention during the third wave due to the higher number of infections). Thus, by analyzing and monitoring IMF$_3$, it is possible to evaluate the performance of public health and social measures and provide alerts on stopping the spread of COVID-19 that capture people’s attention.

Finally, decomposed IMF$_4$ corresponded to the periods of the COVID-19 outbreak in Tokyo. Since mid-February 2020, when people first became more aware of COVID-19 in Japan, there has been dramatic changes in people’s behavior^28^. Gradual and continual public health measures are more effective than one-time government interventions^29^. Thus, in Tokyo, a novel lifestyle with COVID-19 has started under public health measures, such as outdoor activity restrictions at schools and universities and the cancellation of public events^30^. As a result, infection waves occur periodically. These periodical waves are reflected in IMF$_4$, indicating that the period of the COVID-19 outbreak and retreat occurs periodically in Tokyo, 57 days on average. Thus, by analyzing and monitoring IMF$_4$, it is possible to provide a quantitative indicator to help guide decision-making about the duration of states of emergency for future outbreaks in Tokyo.

## Conclusion

The spread of COVID-19 has been studied from various perspectives. A specific epidemiological explanation as to why the COVID-19 outbreak in Tokyo can be controlled periodically remains unknown. Thus, this paper focused on daily COVID-19 infections in Tokyo and provided a nonlinear frequency analysis to demonstrate the effects of social activities during the outbreak of COVID-19 in Tokyo using EMD. The conclusions of our research are as follows:

- Empirical mode decomposition can be considered a powerful tool for analyzing the spread of COVID-19. By adopting EMD to decompose daily COVID-19 infections, we can extract meaningful high frequency to low frequency periods in COVID-19 spread. High-frequency waves correspond to variability due to random factors and variations in the number of PCR and antigen inspections. Low-frequency waves correspond to external influences aiming to stop the spread of COVID-19 and the period of the outbreak and retreat.
- High-frequency IMFs, with average periods of 4.5 days and 7.8 days, can be nonlinearly denoised by EMD to visualize and analyze the spread of COVID-19. Compared with the weekly moving average and FT methods, EMD performed better for analyzing the COVID-19 outbreak.
- A low-frequency IMF has an average period of 19 days, representing intervention effects and information effects on the spread of COVID-19 that are related to social activities. Therefore, public health and social measures can be evaluated and visualized quantitatively by analyzing the corresponding IMF.
- A low-frequency IMF has an average period of 57 days, periodically representing the COVID-19 outbreak and retreat. Analyzing the corresponding IMF can provide a quantitative indicator to guide decision-making about the duration of states of emergency.

In this study, we demonstrated that the decomposed nonlinear mode IMF${_3}$, with an average period of 19 days, corresponds to intervention and information effects in time series and can help visualize and evaluate social and public health measures to stop the spread of COVID-19. However, further experiments and evaluations are required before adopting the instantaneous frequency of IMF${_3}$ to improve social and public health measures.

## Data Availability

The datasets generated and analyzed during the current study are available from the corresponding author on reasonable request.
